# Synaptojanin1 deficiency upregulates basal autophagosome formation in astrocytes

**DOI:** 10.1016/j.jbc.2021.100873

**Published:** 2021-06-11

**Authors:** Ping-Yue Pan, Justin Zhu, Asma Rizvi, Xinyu Zhu, Hikari Tanaka, Cheryl F. Dreyfus

**Affiliations:** Department of Neuroscience and Cell Biology, Robert Wood Johnson Medical School, Rutgers University, Piscataway, New Jersey, USA

**Keywords:** autophagy, astrocyte, cell culture, Parkinson disease, GluT-1, AMPK, AMP-activated protein kinase, DMEM, Dulbecco's modified Eagle's medium, HET, heterozygous, IF, immunofluorescence, LC3, microtubule-associated protein 1A/1B-light chain 3, mTORC1, mechanistic target of rapamycin complex 1, PI3P, phosphatidylinositol 3-phosphate, PI4P, phosphatidylinositol 4-phosphate, RC, R839C, RC SJ1, R839C hSynj1-145 kDa, RQ, R258Q, Synj1, Synaptojanin1, SAC1, suppressor of actin 1, WT SJ1, WT hSynj1-145 kDa

## Abstract

Macroautophagy dysregulation is implicated in multiple neurological disorders, such as Parkinson's disease. While autophagy pathways are heavily researched in heterologous cells and neurons, regulation of autophagy in the astrocyte, the most abundant cell type in the mammalian brain, is less well understood. Missense mutations in the *Synj1* gene encoding Synaptojanin1 (Synj1), a neuron-enriched lipid phosphatase, have been linked to Parkinsonism with seizures. Our previous study showed that the *Synj1* haploinsufficient (*Synj1*^+/−^) mouse exhibits age-dependent autophagy impairment in multiple brain regions. Here, we used cultured astrocytes from *Synj1*-deficient mice to investigate its role in astrocyte autophagy. We report that Synj1 is expressed in low levels in astrocytes and represses basal autophagosome formation. We demonstrate using cellular imaging that *Synj1*-deficient astrocytes exhibit hyperactive autophagosome formation, represented by an increase in the size and number of GFP-microtubule-associated protein 1A/1B-light chain 3 structures. Interestingly, *Synj1* deficiency is also associated with an impairment in stress-induced autophagy clearance. We show, for the first time, that the Parkinsonism-associated R839C mutation impacts autophagy in astrocytes. The impact of this mutation on the phosphatase function of Synj1 resulted in elevated basal autophagosome formation that mimics *Synj1* deletion. We found that the membrane expression of the astrocyte-specific glucose transporter GluT-1 was reduced in *Synj1*-deficient astrocytes. Consistently, AMP-activated protein kinase activity was elevated, suggesting altered glucose sensing in Synj1-deficient astrocytes. Expressing exogenous GluT-1 in *Synj1*-deficient astrocytes reversed the autophagy impairment, supporting a role for Synj1 in regulating astrocyte autophagy *via* disrupting glucose-sensing pathways. Thus, our work suggests a novel mechanism for Synj1-related Parkinsonism involving astrocyte dysfunction.

Regulated membrane trafficking is essential for the function of neurons and glia. Autophagy is part of the intricate membrane trafficking network and often known as the self-eating process that maintains cellular homeostasis and copes with energy crisis. The formation of the autophagosome, a double-membraned structure, is typically induced by nutrient deprivation/starvation, and the sequestered cellular components (damaged organelles or protein aggregates) are eventually degraded in the autolysosome. Autophagy dysregulation has been implicated in various neurodegenerative disorders (1, 2), and the impairment in autophagy clearance is thought to contribute significantly to the accumulation of various forms of protein aggregates found in Alzheimer's disease, Parkinson's disease, and Huntington's disease. Emergent evidence suggests that there is a presence of protein aggregates in astrocytes as in neurons (3, 4), which highlights the importance of astrocyte autophagy in disease progression (5, 6). While autophagy regulation has been studied mostly in heterologous cells and neurons, the process is not well understood in astrocytes, and its distinctive regulatory mechanisms have only begun to be recognized.

Synaptojanin1 (Synj1) is one of the key proteins involved in cellular trafficking. For the past 2 decades, the best-known function of Synj1 has been to facilitate neuronal synaptic vesicle recycling primarily through regulating the conversion of membrane phosphoinositide (7, 8, 9, 10). Synj1 contains two highly conserved inositol phosphatase domains: the suppressor of actin 1 (SAC1)-like domain hydrolyzes phosphatidylinositol 4-phosphate (PI4P) (11) as well as the 3' phosphate on phosphatidylinositol 3-phosphate (PI3P) and PI(3,5)P_2_ (12), whereas the 5'-phosphophatase domain is a more potent enzyme that hydrolyzes the 5' phosphate on the PI(4,5)P_2_ and PI(3,4,5)P_3_. The proline-rich domain of synj1 is more variable and subject to active phosphorylation and protein interaction (13, 14, 15, 16). Variation in the proline-rich domain results in two Synj1 isoforms (17). The 170-kDa long isoform is ubiquitously expressed, whereas the 145-kDa short isoform is known to be enriched in neurons, particularly at the presynaptic terminals and is involved in synaptic vesicle recycling.

Deletion of *Synj1* results in an accumulation of clathrin-coated vesicles at the presynaptic terminal and produces a lethal phenotype at birth (9, 10). In the recent decade, multiple neurological disorders have been linked to the dysregulation of the *SYNJ1* gene. For example, *SYNJ1* gene triplication or overexpression leads to early endosome enlargement, hippocampal dysfunction, and cognitive impairments, which may contribute to early onset Alzheimer's disease and Down syndrome (18, 19, 20, 21). On the other hand, missense mutations in both the SAC1 and 5'-phosphophatase domains of *SYNJ1* have been found to associate with families of early onset atypical Parkinsonism with seizure (22, 23, 24). The Parkinsonism-linked R258Q mutation abolishes the SAC1 activity by ∼80% (22, 25) and is associated with dystrophic changes in both gamma-Aminobutyric acid inhibitory and dopaminergic synapses in mice (25, 26). The R839C mutation, which results in similar clinical phenotypes, has a more profound impact on both phosphatases as it reduces the 5’-phosphatase activity by ∼60% and PI4P hydrolysis by 80% (25). The functional relevance of the R839C mutation, however, has not been explored.

Despite the well-known role of Synj1 in synaptic function, a few recent studies (including one from our laboratory) also suggest its involvement in autophagy regulation (12, 25). Flies carrying the *Synj* R258Q mutation exhibit an impairment in autophagosome maturation at the neuromuscular junction. In the aged *Synj1*^+/−^ mouse brain lysate (25), we found an increase in lipidated microtubule-associated protein 1A/1B-light chain 3 (LC3-II), a hallmark of mature autophagosomes, as well as an increase in the autophagy substrate, p62, indicating an impairment in autolysosomal clearance. However, it was left unclear how astrocytes might have contributed to this pathology. Whether Synj1 regulates astrocyte function remains largely unknown, except for an earlier study that showed its potential contribution to astrogliogenesis (27). Our current study using cultured astrocytes from *Synj1* littermate mice demonstrates that the neuronal isoform, Synj1-145 kDa, is also expressed in the astrocyte. More importantly, we show that endogenous Synj1 represses astrocyte autophagy at the basal level. *Synj1* deletion or the R839C mutation with a complex defect in the SAC1 and 5'-phosphatase activities leads to enhanced autophagosome formation at the basal level. We demonstrate that the role of Synj1 in phosphatidylinositol phosphate metabolism is important for maintaining a proper basal autophagy level, and that the enhanced basal autophagy in *Synj1*-deficient conditions may be related to glucose starvation because of the lack of membrane glucose transporter.

## Results

### Synj1 is expressed in astrocytes and affects the autolysosomes

**Figure 1:**
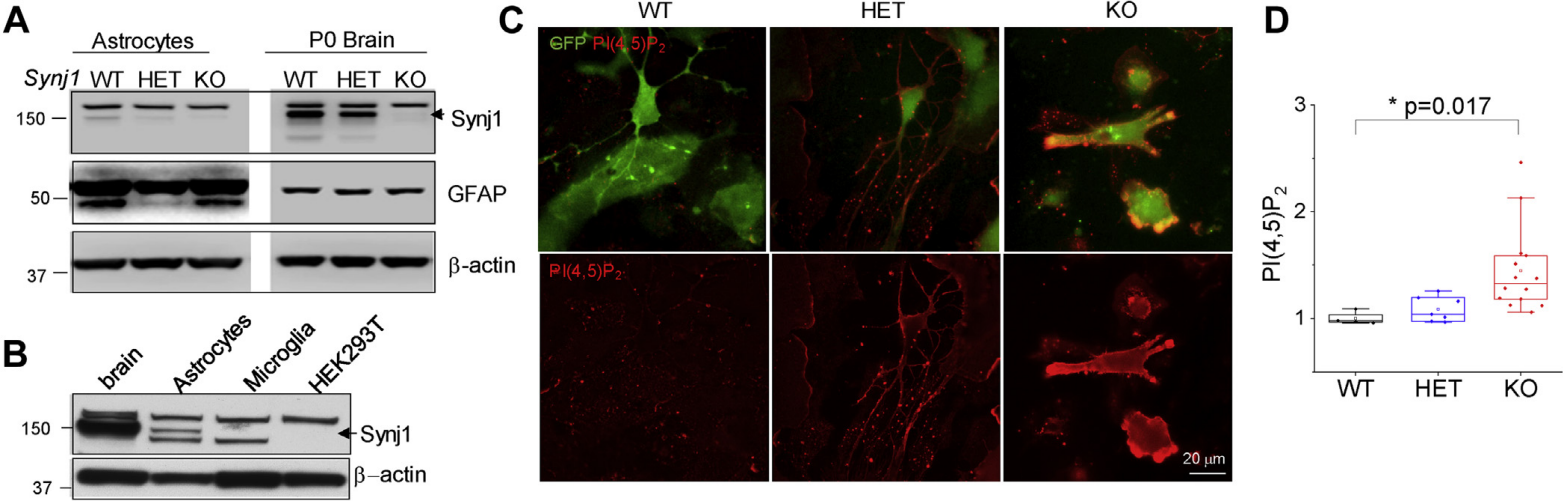
***Synj1* is expressed in the astrocytes and regulates astrocyte membrane P(4,5)P**_**2**_**.***A*, the NBP1-87842 rabbit anti-*Synj1* from Novus Biologicals recognizes the 145-kDa isoform of Synj1, which was abundantly expressed in the brain and weakly expressed in the astrocytes. *White margin* in the *black box* indicates the splicing border from the same membrane. *B*, Western blot analysis of Synj1 expression in adult mouse brain lysate, astrocyte lysate, microglia lysate, and HEK297T cell lysate as indicated using the NBP1-87842 polyclonal antibody. *C*, immunofluorescence for PI(4,5)P_2_ in *Synj1* WT, HET, and KO astrocytes sparsely transfected GFP-LC3. The PI(4,5)P_2_ antibody was validated in our previous report (25). Membrane selections used for PI(4,5)P_2_ analysis. *D*, analysis of the membrane PI(4,5)P_2_ by tracing the contour of the transfected astrocytes shown in (*C*). *p* Value is from Tukey's post hoc test following one-way ANOVA. HEK297T, human embryonic kidney 297T; HET, heterozygous; *Synj1*, Synaptojanin1.

The role of Synj1 in astrocyte is poorly characterized. Using a Novus antibody that specifically recognizes the Synj1-145 kDa (Fig. 1*A*), we identified a low-level expression of neuronal isoform in Synj1 in the cultured cortical astrocytes but not in microglia or human embryonic kidney 293T cells (Fig. 1*B*). Similar to our findings in the cultured cortical neurons (25), deletion of *Synj1* resulted in increased plasma membrane PI(4,5)P_2_, suggesting a prominent role of the 5’-phosphatase domain in astrocyte membrane signaling (Fig. 1, *C* and *D*).

**Figure 2:**
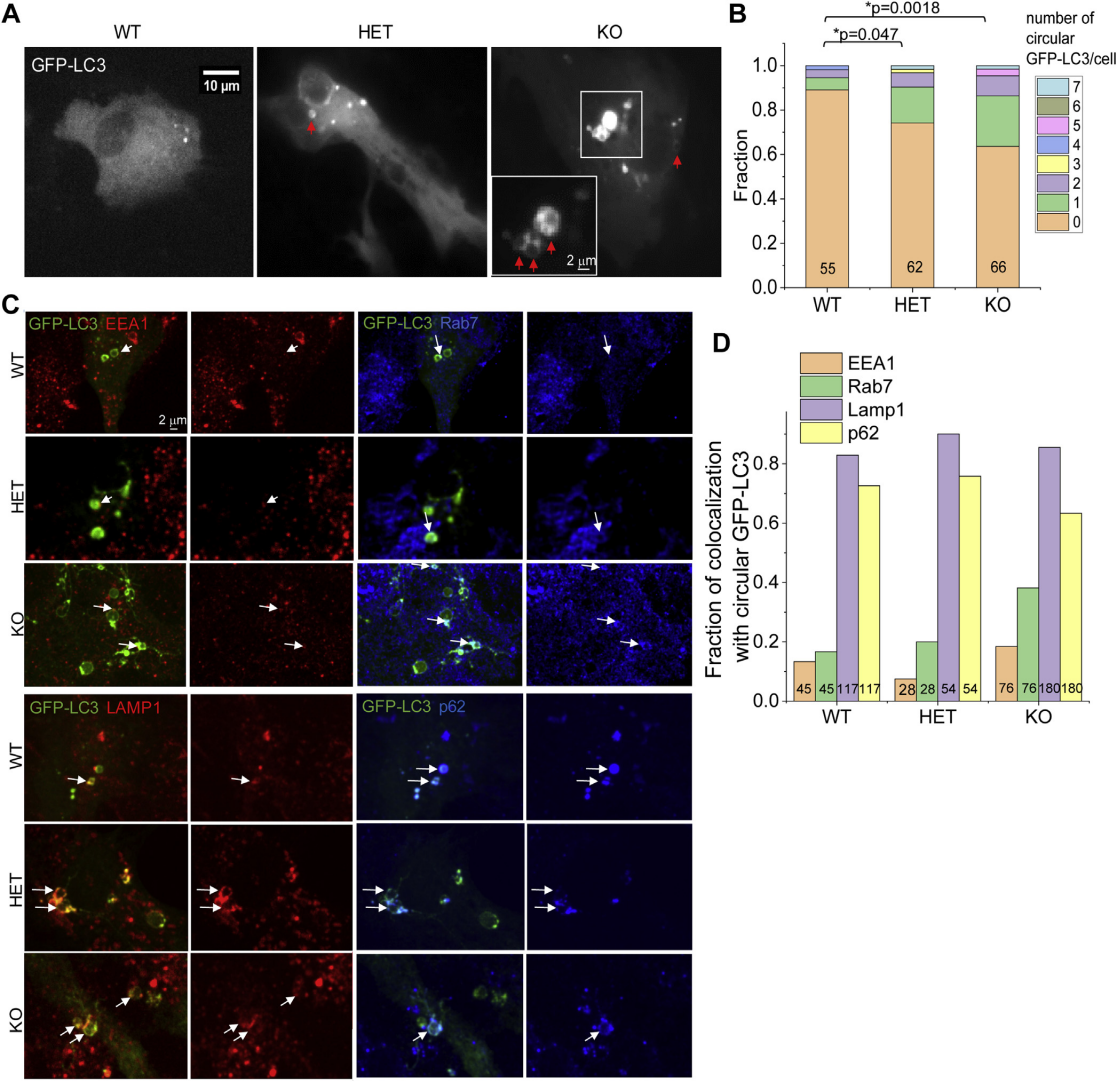
**Increased presence of large autolysosomes in *Synj1*-deficient astrocytes.***A*, cultured astrocytes from *Synj1* WT, HET, and KO brains expressing GFP-LC3. *Arrows* point to circular LC3 structures. *B*, analysis of the occurrence of circular GFP-LC3 structures in littermate astrocyte cultures presented by the stacked column plot. The number of cells analyzed from each genotype was indicated on the column. Data from three independent batches of culture. *p* Values are from the Mann–Whitney *U* test. *C*, WT, HET, and KO astrocytes were transfected with GFP-LC3 and immunolabeled with anti-GFP, EEA1, and Rab7 or anti-GFP, LAMP1, and p62 in separate experiments. *Arrows* point to circular GFP-LC3 structures, which colocalize with one of the protein markers. *D*, bar plot for the fraction of circular GFP-LC3 structure that colocalizes with EEA1, Rab7, LAMP1, or p62. The total numbers of GFP-LC3 structures analyzed in each category were indicated. Data from three batches of WT culture, two batches of HET culture, and three batches of KO culture. HET, heterozygous; *Synj1*, Synaptojanin1.

Autophagy as an important aspect of membrane trafficking has not been well understood in astrocytes. In the *Synj1*^+/−^ mouse, the LC3 immunofluorescence (IF) was not changed in the dopamine neurons; however, a significant increase was noted in the striatum and the cortex, suggesting cell type–specific heterogeneity of the autophagy response to *Synj1* deficiency. To further elucidate the Synj1-mediated autophagy in astrocytes, we expressed GFP-LC3 in cultured astrocytes. In astrocytes prepared from *Synj1*^+/+^ (WT), *Synj1*^+/−^ (heterozygous [HET]), and *Synj1*^−/−^ (KO) littermate pups, we observed LC3 puncta of varying sizes, some of which are shown as circular structures with a hollow center that measured ∼1 to 2 μm in diameter (Fig. 2*A*). We quantified the number of these circular LC3 structures in all GFP-LC3 expressing astrocytes from three batches of primary littermate cultures and found a reverse gene dose dependence in its occurrence (Fig. 2*B*). To determine the nature of these organelles, we performed additional immunolabeling analyses to assess their colocalization with the early endosome marker, EEA1; the late endosome marker, Rab7; the lysosomal marker, LAMP1; as well as the autophagy adaptor, p62 (Fig. 2*C*). We found that, in all astrocytes regardless of the Synj1 level, the majority of the circular GFP-LC3 structures colocalize with LAMP1 (WT: 82.9%, HET: 90%, and KO: 85.5%) and p62 (WT: 72.6%, Het: 75.8%, and KO: 63.3%). A smaller fraction of these structures was found to colocalize with EEA1 (WT: 13.3%, HET: 7.5%, and KO: 18.4%) or Rab7 (WT: 16.7%, HET: 20%, and KO: 38.2%) (Fig. 2*D*). Our data suggest that the abnormal accumulation of circular GFP-LC3 structures in *Synj1* deficiency astrocytes represents a dysregulated autolysosomal pathway.

### Synj1 deficiency enhances the basal autophagosome formation in astrocytes but impairs autophagy clearance

**Figure 3:**
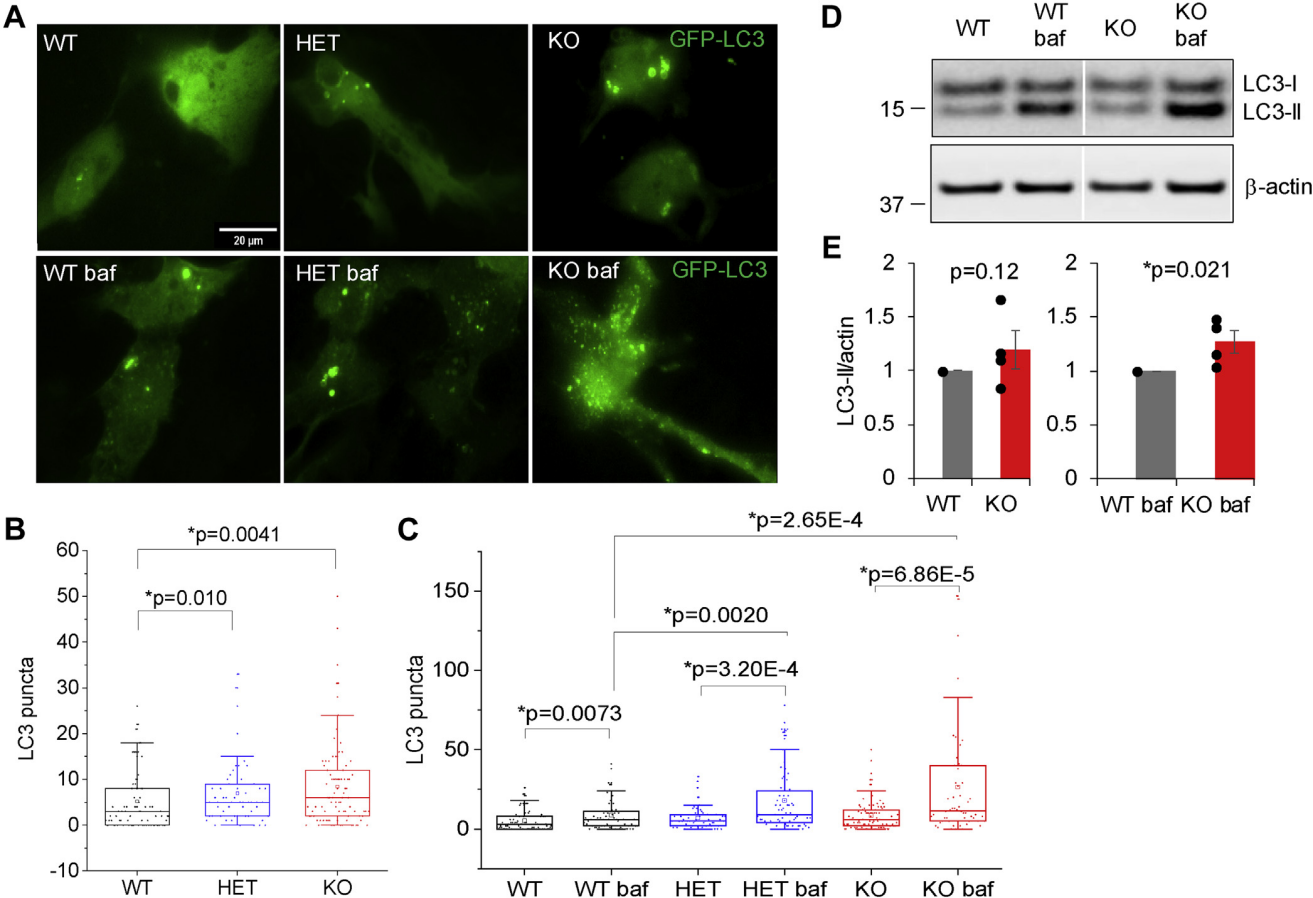
**The basal autophagosome formation is enhanced in *Synj1*-deficient astrocytes.***A*, representative images of cultured astrocytes from P0 *synj1* WT, HET, and KO brains expressing GFP-LC3 (*upper panels*) and those that treated with 20 nM bafilomycin A1 for 1 h (*lower panels*). The ctrl HET image is reused from Figure 2*A*. *B* and *C*, box plots comparing the number of LC3 puncta at the basal level (*B*) and those after the treatment of bafilomycin A1 in GFP-LC3 expressing cells (*C*). Data from four independent batches of cell cultures. *p* Values in (*B*) and (*C*) are from Mann–Whitney *U* tests. *D* and *E*, Western blot analysis for cultured WT and KO astrocytes at baseline and those that were treated with bafilomycin A1 for 1 h. *White margin* in the *black box* (*D*) is the splicing border from the same membrane. Data from four batches of cells and *p* values are from Student's *t* test. HET, heterozygous; *Synj1*, Synaptojanin1.

We next analyzed the number of GFP-LC3 puncta in cultured astrocytes. In *Synj1* HET or KO astrocytes, the GFP-LC3 puncta was modestly but significantly higher (HET: 6.95 ± 0.92, N = 57; KO: 8.46 ± 1.00, N = 91; four batches) compared with that in the WT littermates (5.19 ± 0.73, N = 81; four batches) (Fig. 3, *A* and *B*). In a separate set of littermate astrocytes, where bafilomycin A1 (baf, 20 nM) was applied for an hour to inhibit autolysosomal degradation, a much more striking increase was observed for *Synj1*-deficient astrocytes (HET: 18 ± 2.54, N = 75 and KO: 26.50 ± 4.49, N = 58; four batches) compared with WT (8.74 ± 1.22, N = 62; four batches) (Fig. 3, *A* and *C*). Consistently, our Western blot analysis also showed significant accumulation of lipidated LC3-II after bafilomycin treatment (Fig. 3, *D* and *E*). These results, taken together, suggest hyperactive formation of autophagosome in *Synj1*-deficient astrocytes at the basal level (Fig. 3).

**Figure 4:**
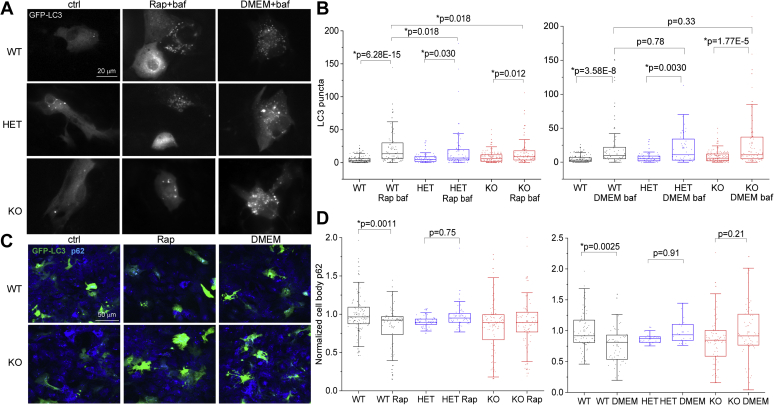
***Synj1* deficiency impairs autophagy clearance.***A*, representative images of cultured astrocytes from P0 *synj1* WT, HET, and KO brains expressing GFP-LC3 (ctrl), those that were treated with 200 nM rapamycin (Rap + baf) or the DMEM-only medium (DMEM + baf) for 4 h including a 20 nm bafilomycin A1 treatment in the last hour. WT and HET ctrl images were reused from Figure 2*A*. *B*, box plots comparing the number of LC3 puncta between the ctrl and the rapamycin-treated groups (*left*) or between ctrl and the DMEM-only medium treated groups (*right*). *p* Values were from Mann–Whitney *U* tests. *C*, representative images of cultured astrocytes from P0 *synj1* WT and KO brains expressing GFP-LC3 (ctrl), those that were treated with 200 nM rapamycin for 4 h (Rap), and those that were treated with the DMEM-only medium for 4 h. Cells were fixed simultaneously and immunolabeled with GFP and p62. *D*, box plots comparing the normalized p62 levels across in the ctrl and the rapamycin-treated groups (*left*) or between ctrl and the DMEM-only medium treated groups (*right*). *p* Values are from Tukey's post hoc test following two-way ANOVA. Data from four independent batches of cell cultures. DMEM, Dulbecco's modified Eagle's medium; HET, heterozygous; *Synj1*, Synaptojanin1.

We next examined how effective *Synj1*-deficient astrocytes are in clearing autophagy substrates. In cultured WT neurons from the murine models, starvation or pharmacologically inhibiting the mechanistic target of rapamycin complex 1 (mTORC1) activity with rapamycin or torin are usually ineffective in inducing autophagic clearance (28). Our previous study, however, showed an intriguing sensitization of the *Synj1*^+/−^ dopamine neurons to rapamycin-induced p62 clearance (25). To examine the stress-induced autophagy in astrocytes, we used two strategies: a 4-h rapamycin (200 nM) treatment to inhibit mTORC1 (29, 30) or serum starvation (replacing the culture medium containing 10% fetal bovine serum to the Dulbecco's modified Eagle's medium [DMEM] only medium) to mimic nutrient deprivation. WT astrocytes responded robustly and consistently to rapamycin and serum deprivation with an increase in GFP-LC3 puncta (control: 4.75 ± 0.53, N = 120; Rap: 22.72 ± 2.78, N = 82, DMEM: 23.22 ± 4.10, N = 70) (Fig. 4, *A* and *B*) and a reduction in p62 IF (normalized control: 1 ± 0.025, N = 109; normalized Rap: 0.83 ± 0.031, N = 82; normalized DMEM: 0.76 ± 0.041, N = 57) (Fig. 4, *C* and *D*) in all four batches of cultures examined. However, rapamycin was ineffective in clearing the autophagy substrate, p62, in both HET and KO astrocytes. Despite a weaker LC3 response to rapamycin in *Synj1* KO astrocytes, their responses to the 4-h serum deprivation were as robust as the WT cells (KO DMEM: 28.41 ± 4.52, N = 78, HET DMEM: 21.10 ± 3.31, N = 50) (Fig. 4, *A* and *B*, *right panel*). The p62 level, however, remained unaffected. Taken together, our data suggest that *Synj1* deficiency impairs autolysosomal degradation in response to stress (Fig. 4).

### The Synj1 phosphatase domains play a major role in regulating astrocyte autophagosome formation

**Figure 5:**
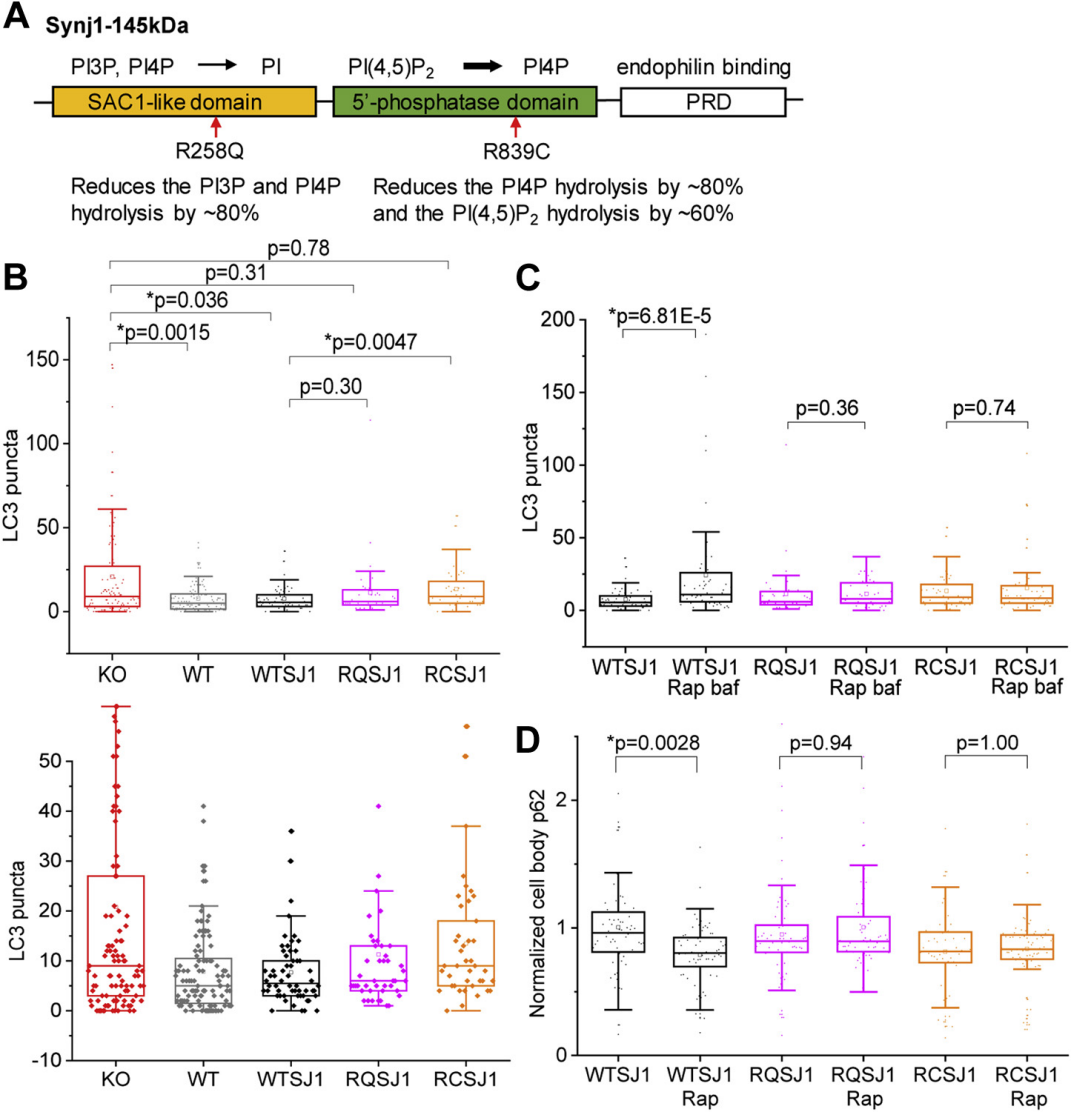
**The Synj1 phosphatase domains play a major role in regulating astrocyte autophagosome formation.***A*, domain structure and function of Synj1 with *arrows* pointing to the known Parkinsonism mutations illustrated by the functional outcome of the mutations reported in our previous publication (25). *B*–*D*, WT hSynj1 (WTSJ1), R258Q hSynj1 (RQSJ1), or R839C hSynj1 (RCSJ1) were expressed in the *Synj1* KO astrocytes and compared with the KO and its littermate WT astrocyte culture. *B*, box plots comparing the number of LC3 puncta at the basal level with 1-h baf treatment for all groups. *C* and *D*, box plots comparing the rapamycin-induced autophagy markers, the number of GFP-LC puncta (*C*) and p62 (*D*) in KO cells expressing WT hSynj1 (WTSJ1), R258Q hSynj1 (RQSJ1), or R839C hSynj1 (RCSJ1). *p* Values for the LC3 analyses are from Mann–Whitney *U* tests. *p* Values for p62 analyses are from Tukey's post hoc tests following two-way ANOVA. *Synj1*, Synaptojanin1.

Multiple Parkinsonism-related *Synj1* mutations have been identified (22, 23, 24, 31), and all mutations reside in the two phosphatase domains (Fig. 5*A*). Our previous study using an *in vitro* phosphatase assay showed that the disease-linked R258Q (RQ) mutation abolishes the PI4P and PI3P hydrolysis by ∼80% (22, 25), whereas the R839C (RC) mutation reduces the 5’-phosphatase activity by ∼60% and PI4P hydrolysis by 80% (25). To date, studies of the RQ mutation have suggested its role in maintaining the axonal and synaptic morphology (26), autophagosome maturation (12), and endosomal trafficking (32); however, the functional relevance of the RC mutation has not been documented (Fig. 5).

We expressed GFP-LC3 with either the RQ hSynj1-145 kDa, RC hSynj1-145 kDa (RC SJ1) or WT hSynj1-145 kDa (WT SJ1) in the *Synj1* KO astrocytes and analyzed how these mutations affect autophagosome formation by comparing with the littermate WT cells and KO cells. We found that the WT SJ1 expression effectively reversed the increased GFP-LC3 puncta number in KO astrocytes (KO: 20.78 ± 2.85, N = 105 compared with WT SJ1 rescue: 7.69 ± 0.90, N = 58 and WT littermate: 7.81 ± 0.79, N = 112; three batches) (Fig. 5*B*). However, the RC SJ1 was unable to repress the increased autophagosomes in the KO background (RC SJ1: 13.44 ± 1.94, N = 41, compared with KO: 20.78 ± 2.85, N = 105, *p* = 0.78 Mann–Whitney *U* test) (Fig. 5*B*). The rescue efficiency for the RQ hSynj1-145 kDa was in between the WT SJ1 and RC SJ1. The LC3 puncta numbers were different from neither the KO cells nor the WT SJ1 rescue (Fig. 5*B*). Our data suggest that both phosphatase activities of Synj1 are important for regulating basal level autophagy, but the RC mutation with a more profound defect in the phosphatase activities could produce a phenotype that mimics *Synj1* deletion.

Because of concerns of SJ1 overexpression in these experiments, we performed a correlation analysis for Synj1 IF and GFP-LC3 puncta. Our data showed that in the subset of cells where the exogenous WT SJ1 was expressed at the endogenous level (determined by the WT littermate culture), the rescue for GFP-LC3 puncta was equally effective (Fig. S1). Overexpressing WT SJ1 did not lead to significant changes neither in the LC3 puncta numbers nor the SJ1 mutants.

We next examined how the Synj1 disease mutants affected the mTORC1 regulated autophagy. Not surprisingly, WT SJ1 was able to restore the rapamycin sensitivity in autophagosome formation (WT SJ1: 7.69 ± 0.90, N = 58; WT SJ1 Rap: 24.29 ± 4.78, N = 59; three batches) and p62 clearance (WT SJ1: 1.00 ± 0.053, N = 66; WT SJ1 Rap normalized to WT SJ1: 0.79 ± 0.029, N = 70; three batches), but neither of the SJ1 mutants did (Fig. 5, *C* and *D*).

### Altered glucose sensing in Synj1-deficient astrocytes contributes to hyperactive autophagosome formation

**Figure 6:**
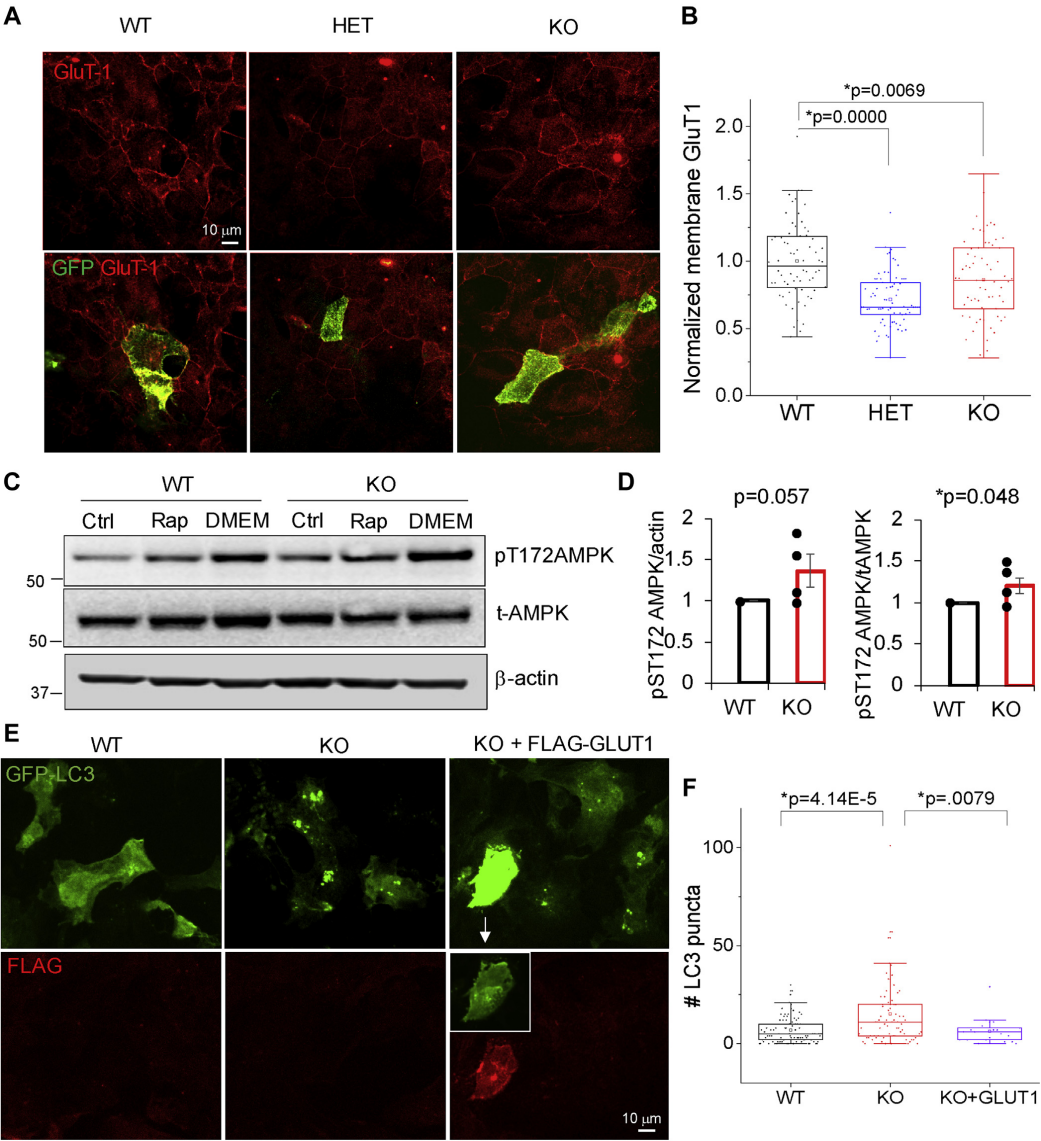
**Altered glucose sensing contributes to the hyperactive autophagosome formation in *Synj1*-deficient astrocytes.***A*, representative images of cultured astrocytes from P0 *synj1* WT, HET, and KO brains expressing GFP and immunolabeled with anti-GluT-1. *B*, box plots for membrane immunofluorescence of GluT-1 in cultured astrocytes. Data from three independent cultures. *p* Values were from Tukey's post hoc tests following one-way ANOVA. *C* and *D*, representative Western blots for WT and KO astrocytes at the basal condition (ctrl) as well as those that treated with rapamycin (Rap) or serum starvation (DMEM) for 4 h (*C*). Analysis for AMPK activity and expression in four independent cultures at the basal level. *p* Values are from Student's *t* test. *E*, representative images of cultured astrocytes from P0 *synj1* WT and KO brains sparsely expressing GFP-LC3 or GFP-LC3 with FLAG-GLUT1. Cells were fixed and immunolabeled with anti-GFP and FLAG. *White box* indicates the contrast-adjusted GFP-LC3 signal in the GLUT1 expressing cell. *F*, box plots for the number of GFP-LC3 puncta in WT, KO, and KO + GLUT1 astrocytes. *p* Values are from Tukey's post hoc tests following one-way ANOVA. AMPK, AMP-activated protein kinase; DMEM, Dulbecco's modified Eagle's medium; HET, heterozygous; *Synj1*, Synaptojanin1.

To understand the molecular pathways that may underlie the enhanced basal level autophagosome formation in *Synj1*-deficient astrocytes, we analyzed the expression of an astrocyte-specific glucose transporter, GluT1. A previous report showed that the GluT1 level was reduced in the *Synj1*^−/−^ mice (27). Our Western blot analysis for cultured astrocytes from *Synj1*^−/−^ mice found varying levels of GluT1 relative to *Synj1*^+/+^ astrocytes (data not shown); however, a consistent and significant reduction of GluT1 was observed at the membrane of both *Synj1*^+/−^ and *Synj1*^−/−^ astrocytes across three independent batches of cultures (Fig. 6, *A* and *B*). Consistently, the AMP-activated protein kinase (AMPK) activity, which is often activated in response to glucose starvation (33), was enhanced at the basal level (Fig. 6, *C* and *D*). Thus, our data suggest altered glucose sensing in *Synj1*-deficient astrocytes, which may contribute to the hyperactive autophagosome formation. Indeed, expressing exogenous GluT1 in KO astrocytes rescued the hyperactive formation of autophagosomes at the basal level (Fig. 6, *E* and *F*). Taken together, our study suggests that Synj1 deficiency alters glucose sensing for astrocytes, which results in hyperactive formation of autophagosomes at the basal level (Fig. 6).

## Discussion

Our study demonstrates that the well-characterized neuronal protein, Synj1, is also present in astrocytes and regulates astrocyte PI(4,5)P_2_ metabolism. We further reveal a novel role for Synj1 in repressing astrocyte autophagosome formation at the basal level. *Synj1* deficiency results in an accumulation of abnormally large autolysosomes. The autolysosomal clearance, however, is ineffective in meeting the needs of hyperactive autophagosome formation when external stressors are present. We show that the phosphatase activities of Synj1 are important in keeping the basal level autophagy in check. The Parkinsonism-linked R839C mutation, which has a more profound impact on the phosphatidylinositol phosphate metabolism than the R258Q mutation (25), is reported for the first time to promote basal level autophagosome formation that mimicked *Synj1* deletion. Both the R258Q and the R839C Parkinsonian mutations impaired the rapamycin-induced autophagy in astrocytes, suggesting that the intact phosphatase activities in Synj1 are especially crucial for astrocytes to cope with cellular stress such as nutrient starvation. Moreover, we demonstrate that glucose sensing is altered in *Synj1*-deficient astrocytes, which may, in part, contribute to the hyperactive autophagosome formation at the basal level.

Additional molecular machineries may be engaged during stress-induced autophagy. In an earlier study, it was suggested that PI(3,5)P_2_ accumulation because of the R258Q mutation could impede LC3 lipidation in the *drosophila* neuromuscular junction (12). However, an enhanced basal level autophagy was not reported in the *Synj* mutant *drosophila*. Our finding of hyperactive autophagosome formation in the *Synj1*-deficient cortical astrocytes is reminiscent of our previous observation in the *Synj1*^+/−^ mouse brains (25), where we showed an increase in the endogenous LC3 IF in the cortex and striatum. While we were unable to distinguish an effect from the glia or the neuron, then our current study suggests that the increased LC3 might in part be due to an upregulated autophagosome formation in the astrocytes at the basal level.

Our study suggests that glucose sensing plays a crucial role in regulating the basal level astrocyte autophagy (34, 35, 36). The recycling of the glucose transporter on the plasma membrane is a dynamic and highly regulated process. The role of Synj1 in PIP_2_ hydrolysis and clathrin uncoating may be essential for membrane insertion of the GluT1. A previous study showed that PTEN, a PIP_2_-generating enzyme, prevents endosome-to-plasma membrane recycling of GluT1 (37), reminiscent of *Synj1*-deficient astrocytes. The hypothesis is also partially supported by our Synj1 mutant analysis. The Parkinsonism R839C mutation that disrupts 60% of the 5’-phosphatase activity enhanced autophagosome formation at the basal level, mimicking that of *Synj1* KO astrocytes; whereas the R258Q mutation that does not affect the 5’-phosphatase activity showed minimal impact. However, it remains to be elucidated in greater molecular detail if the accumulation of PI(4,5)P_2_ is the culprit for GluT1 membrane expression and astrocyte energy sensing and whether amino acid transporters are also affected.

As multiple missense mutations in *SYNJ1* were found in Parkinsonian patients with seizure, it remains to be investigated whether and how Synj1 deficiency contributes to the pathogenic course *in vivo*. Interestingly, GLUT1 deficiency has been shown to associate with epilepsy (38, 39), which begs further investigation in astrocyte-mediated pathogenic mechanism. More importantly, is the hyperactive autophagosome formation correlated with an abnormal phagocytic activity in astrocytes that results in dopaminergic synapse pruning (40)? Or will the inflexibility of astrocytes to respond to external stressors result in an accumulation of alpha-synuclein (25) and reactive oxygen species? Astrocytes, as major supporting cells in the brain, are actively involved in secretion of neurotrophic factors and cytokines to regulate neuronal plasticity and neuroinflammation (41, 42). Astrocytes from different parts of the mouse brain have also been shown to perform different roles to support the neuron (43, 44). It remains to be researched if astrocytes from the striatum or the midbrain also exhibit altered autophagy function to ultimately impact dopaminergic neurotransmission and neuronal survival (45).

Despite the conserved pool of autophagy genes from yeast to mammalian cells, tremendous differences exist across species and cell types for its regulatory mechanisms (5, 28, 46, 47). Although neuronal autophagy has been shown to be less influenced by nutrient deficiency (28, 48, 49), it remains to be elucidated if an enhanced basal level autophagy flux is also present in *Synj1*-deficient murine and human neurons. Thus, our study reveals a novel role of Synj1 in astrocyte autophagy/energy sensing and brings insight to Synj1-mediated pathogenic mechanisms.

## Experimental procedures

### Animals

Mice were housed in the pathogen-free barrier facility at the Rutgers Robert Wood Johnson Medical School SPH vivarium. Handling procedures were in accordance with the National Institutes of Health guidelines approved by the Institutional Animal Care and Use Committee. The *Synj1*^+/−^ mice (9) were obtained from the Pietro De Camilli laboratory at Yale University. As the *Synj1*^−/−^ mouse is lethal at birth, *Synj1*^+/−^ mice were used as breeders to generate KO pups and littermates.

### Cell culture and transfection

Astrocyte cultures were prepared from postnatal day 0 (P0) littermate pups of both sexes using a slightly modified protocol from published methods (50). Mice were decapitated by sharp scissors, and the brains were dissected in ice-cold Hank's solution (H2387; Sigma) containing 350 mg/l NaHCO_3_ and 1 mM Hepes (260 mg/l) with pH adjusted to 7.15 to 7.20. Typically, two cortices from mice of each genotype were dissected and broken into smaller pieces by the spring scissors. The tissues were then digested at room temperature for 7 min in a 3-ml Hank's solution containing 0.25% Trypsin (15090046; Thermo Fisher) and 0.1 μg/μl DNase (D5025; Sigma) with intermittent shaking. Tissues were mechanically dissociated by pipetting, and the trypsin reaction was terminated by addition of 4 ml culture media containing DMEM (11965118; Thermo Fisher), 10% fetal bovine serum (S11550; Atlantic Biologicals), and 10 U/ml penicillin-streptomycin (15140122; Thermo Fisher). Cells were centrifuged at 300*g* for 10 min and plated at ∼8,000,000/10 cm dish precoated with poly-d-lysine (A-003-E; Sigma; 0.1 mg/ml). Culture medium was changed every 2 to 3 days after plating, and cells typically reach 90% confluency after 10 days. To obtain an enriched astrocyte culture, the culture dish was placed on an orbital shaker at ∼180 rpm for 30 min to remove microglia (or to obtain a separate microglia culture) and an additional 6 h at ∼240 rpm to remove oligodendrocyte precursor cells (50). The remaining confluent astrocyte culture was rinsed by PBS and digested with 0.05% trypsin-EDTA (25300054; Thermo Fisher). Enriched astrocytes from the first or the second passage were grown on cover glasses (#1.5) for imaging studies or on 6-well plates for Western blot analysis. Human embryonic kidney 293T cells were grown in the same culture media as the astrocyte culture and maintained/passaged using the same procedure. For imaging analysis, cells were plated at 50% confluency and transfected the next day with GFP-LC3 (#21073; Addgene) or double transfected with GFP-LC3 and one of the FLAG-hSynj1 constructs (see later). The Lipofectamine3000 reagent was used for transfection following a company suggested protocol.

### Constructs

pEGFPC1-FLAG-WT *hSYNJ1*-145 kDa, pEGFPC1-FLAG-R258Q *hSYNJ1*-145 kDa, and pEGFPC1-FLAG-R839C *hSYNJ1*-145 kDa (25) were re-engineered by site-directed mutagenesis (200517; Agilent QuikChange) to delete the enhanced GFP using the following primers: 5’-CGCTAGCGCTACCGGTCGCCACCTCCGGACTCAGATC-3’ and 5’-GCTTGAGCTCGAGATCTGAGTCCGGAGGTGGCGACCGG-3’. The successful deletion of the enhanced GFP was verified by sequencing. FLAG-GLUT1 was purchased from Addgene (#89571).

### Western blot analysis and antibodies

Brain samples or cells were lysed on ice for 30 min using a Triton-based lysis buffer containing 50 mM Tris–HCl (pH 7.5), 150 mM NaCl, 1% Triton, as well as protease and phosphatase inhibitors as previously described (16, 25). After centrifugation at 16,000*g*, 4 °C for 30 min, the supernatant was collected for protein quantification using the Pierce bicinchoninic acid assay (23227; Thermo). Typically, 5 to 10 μg of total proteins were loaded for each sample on the Invitrogen 4 to 12% Bis–Tris gel, and the following antibodies were used for immunoblot detection: rabbit anti-Synj1 (NBP1-87842; Novus Biologicals; 1:1000), rabbit anti-GFAP (A0237; Abclonal; 1:2000), rabbit anti-pT172 AMPK (2535; Cell Signaling; 1:1000), rabbit anti-AMPKα (2532; Cell Signaling; 1:1000), mouse anti-β-actin (37005; Cell Signaling; 1:3000). All Western blots were performed with two to three technical repeats, and the Western blot bands were analyzed using ImageJ (Rasband, W.S., ImageJ, U. S. National Institutes of Health, https://imagej.nih.gov/ij/).

### IF analysis

The following antibodies were used for IF: mouse anti-PI(4,5)P_2_ (z-P045; Echelon Biosciences; 1:100) (25), chicken anti-GFP (A-10262; Thermo Fisher; 1:1000), guinea pig anti-p62 (GPP62-C; Progene; 1:1000), rabbit anti-EEA1 (ab109110; Abcam; 1:1000), mouse anti-Rab7 (ab50533; Abcam; 1:200), rat anti-LAMP1 (14-1071-85; Invitrogen; 1:1000), rabbit anti-GLUT1 (A6982; Abclonal; 1:100), and mouse anti-FLAG (F1804; Sigma; 1:500). Immunocytochemistry was performed following previously published procedures (16, 25, 51). Immunofluorescence was analyzed using a Nikon Ti-2 wide-field microscope with Spectra-X (Lumencor) as the light source and an Andor Ultra 897 EMCCD camera. The Alexa Fluor-488, Alexa Fluor-546, and Alexa FluoR-647 emissions were collected using the ET535/50m, ET585/40m, and ET665lp emission filters, respectively. All imaging parameters were set to the same for each batch of culture. Image stacks were taken at different focal planes at 0.9-μm interval to include the whole cell, and a maximum projection image was generated for each stack *via* ImageJ for analysis. All analyses were done manually. The GFP-LC3 punctum was determined by 1.5 × 1.5 μm (6× 6 pixels) circular regions of interests, which means larger punctum was counted as multiple puncta. The p62 level was analyzed as whole cell IF in randomly transfected GFP-LC3 cells. The circular GFP-LC3 colocalization analysis (Fig. 2) and GluT1 IF analysis (Fig. 6) were performed using a Nikon CREST spinning disk confocal microscope with a 100× oil emersion objective (numerical aperture = 1.45).

### Data analysis

All imaging and Western blot studies were from at least three independent primary cultures. Most Western blots were repeated three times. The number of GFP-LC3 puncta at the basal level remained consistent in all batches. The p62 IF and protein expression levels in Western blots varied across different batches and were all normalized to the values obtained in the WT cells in the same batch. The LC3 quantification data do not follow normal distribution, and the statistical difference was calculated using Mann–Whitney *U* test. For datasets following normal distribution, Student's *t* test or one-way/two-way ANOVA was performed followed by Tukey's post hoc analysis using the build-in functions in OriginLab.

## Data availability

All data and plasmids described in the article are available for sharing upon request.

## Supporting information

This article contains supporting information.

## Conflict of interest

The authors declare that they have no conflicts of interest with the contents of this article.
