# Supplementary material for: Synaptojanin1 deficiency upregulates basal autophagosome formation in astrocytes
Source: J Biol Chem. 2021 Jun 11;297(1):100873. doi: 10.1016/j.jbc.2021.100873 (PMC8258991; doi:10.1016/j.jbc.2021.100873)
Supplement: Supplemental Figure S1 [file mmc1.pdf]

Supporting information for

Synaptojanin1 deficiency upregulates basal autophagosome formation in astrocytes

Authors: Ping-Yue Pan*, Justin Zhu, Asma Rizvi, Xinyu Zhu, Hikari Tanaka, Cheryl F. Dreyfus

Author affiliation: Department of Neuroscience and Cell Biology, Robert Wood Johnson Medical School, Rutgers University, Piscataway, NJ 08854 USA

***correspondence**: Ping-Yue Pan, Ph.D. email: pingyue.pan@rutgers.edu, address: 675 Hoes Ln, Research Tower R330, Robert Wood Johnson Medical School, Rutgers University, Piscataway, NJ 08854 USA

Running title: Synaptojanin1 in astrocyte autophagy

Key words: Autophagy, astrocyte, cell culture, Parkinson disease, GluT-1

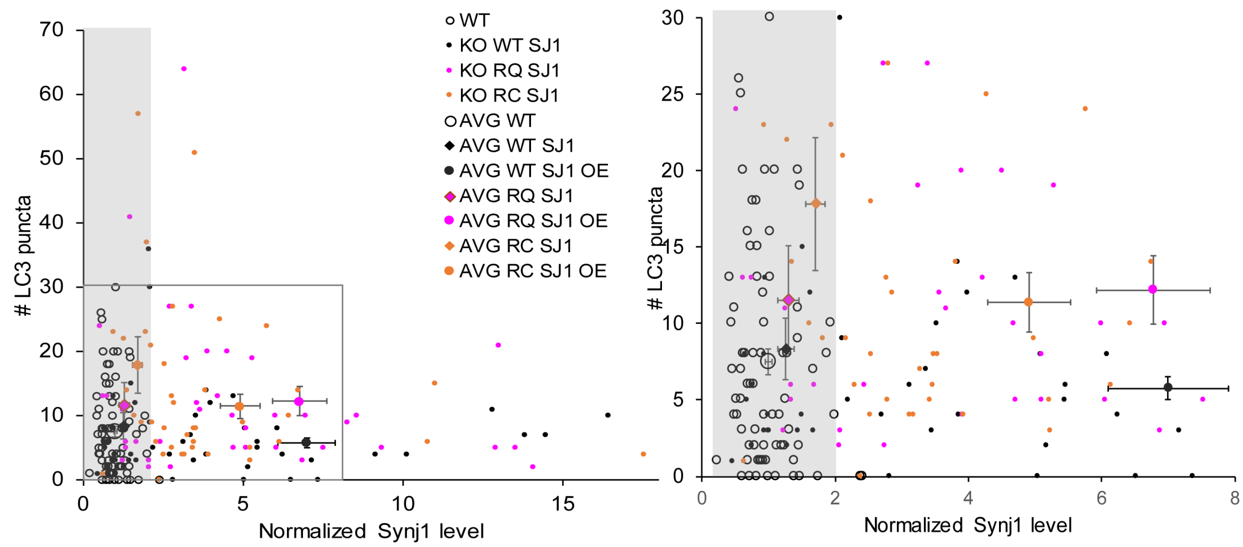

Fig. S1: Synj1 overexpression does not affect astrocyte autophagosome

Two-dimensional plot for the correlation between the number of GFP-LC3 puncta and the Synj1 expression level. Data were extrapolated from Fig. 4. Synj1 expression levels measured in the WT cells were considered physiological levels of Synj1 (normalized to 1) with ranges indicated by the shaded grey boxes. The highest physiological level of Synj1 in WT cells divides each dataset (either expressing WT, RQ or RC synj1) into a rescue/replacement group (KO WT SJ1, KO RQ SJ1 or KO RC SJ1) and an overexpression (OE) group WT SJ1 OE, RQ SJ1 OE, or RC SJ1 OE). Each small symbol represents a measurement from a cell, and large symbols with error bars were averages from either the rescue/replacement or OE group. Two-way ANOVA for protein expression and genotype followed by *post hoc* didn't reveal any significant difference. Neither did the correlation tests.
